# MCSF drives regulatory DC development in stromal co-cultures supporting hematopoiesis

**DOI:** 10.1186/s12865-018-0255-y

**Published:** 2018-06-26

**Authors:** Sawang Petvises, Pravin Periasamy, Helen C. O’Neill

**Affiliations:** 1 Division of Biomedical Science, Research School of Biology, The Australian National University, Canberra, Australia; 2 Department of Medical Technology, Faculty of Applied Health Sciences, Thammasat University, Bangkok, Thailand; 3 Department of Microbiology, Yong Loo Lin School of Medicine, National University of Singapore, Singapore, Singapore; 4 Clem Jones Centre for Regenerative Medicine, Faculty of Health Sciences and Medicine, Bond University, Gold Coast, QLD Australia

**Keywords:** Hematopoiesis, Dendritic cell, Regulatory dendritic cells, Regulatory T cells

## Abstract

**Background:**

Splenic stroma overlaid with hematopoietic progenitors supports in vitro hematopoiesis with production of dendritic-like cells. Co-cultures of murine lineage-depleted bone marrow over the 5G3 stromal line produce two populations of cells, characterised as CD11b^+^CD11c^+^MHC-II^−^ dendritic-like ‘L-DC’, and CD11b^+^CD11c^+^MHC-II^+^ cells, resembling conventional dendritic cells (cDC). To date, the functional capacity of these two subsets has not been clearly distinguished.

**Results:**

Here we show both the L-DC and cDC-like subsets can be activated and induce proliferation of OT-I CD8^+^ T cells, being strong inducers of IL-2 and IFN-γ production. Both subsets lack ability to induce proliferation of OT-II CD4^+^ T cells. The cDC-like population is shown here to resemble regulatory DC in that they induce FoxP3 expression and IL-10 production in OT-II CD4^+^ T cells, in line with their function as regulatory DC. L-DC did not activate or induce the proliferation of CD4^+^ T cells and did not induce FoxP3 expression in CD4^+^ T cells. L-DC can be distinguished from cDC-like cells through their superior endocytic capacity and expression of 4-1BBL, F4/80 and Sirp-α. A comparison of gene expression by the two subsets was consistent with L-DC having an activated or immunostimulatory DC phenotype, while cDC-like cells reflect myeloid dendritic cells with inflammatory and suppressive properties, also consistent with functional characteristics as regulatory DC. When a Transwell membrane was used to prevent hematopoietic cell contact with stroma, only cDC-like cells and not L-DC were produced, and cell production was dependent on M-CSF production by stroma.

**Conclusion:**

Co-cultures of hematopoietic progenitors over splenic stroma produce two distinct subsets of dendritic-like cells. These are here distinguished phenotypically and through gene expression differences. While both resemble DC, there are functionally distinct. L-DC activate CD8^+^ but not CD4^+^ T cells, while the cDC-like population induce regulatory T cells, so reflecting regulatory DC. The latter can be enriched through Transwell co-cultures with cell production dependent on M-CSF.

## Background

Dendritic cells (DC) are potent antigen presenting cells (APC) essential for activation of naïve T cells [1]. While DC are clearly important in activation of T cell immunity, they also play an important role in immune tolerance and homeostasis through suppression of T cell activation and function [2, 3]. While immune activating DC are typified by the main splenic subsets of conventional (c) and plasmacytoid (p) DC, tolerising or regulatory DC (DCregs) are less well defined, with heterogeneity already reported within the field [4]. In general, DCregs are dendritic-like cells distinct by their CD11b^hi^CD11c^lo^ phenotype with low expression of both MHC-II and costimulatory molecules like CD40, CD80 and CD86 [5]. The important involvement of DCregs in controlling autoimmunity and allograft rejection can be attributed to their specific ability to induce the production of FoxP3-expressing regulatory T cells (Tregs) which suppress activation of antigen-specific T cells [4, 5]. The therapeutic importance of this DC subset has led to a number of studies on the production of DCregs in vitro with a view to their use in cell therapy [4, 6].

The importance of the stromal microenvironment in production of DCregs has been demonstrated for multiple tissues including spleen, liver, lung and even tumours [7–11]. Several reports indicate production of DCregs in vitro through co-culture of bone marrow above splenic stromal cells [9, 11–13]. However, the stromal cell contribution to DCreg production, and the pathway to development are not well characterised. Previous studies from this lab have shown that continuous longterm spleen stromal cultures support production of distinct CD11b^hi^CD11c^lo^ dendritic-like cells lacking MHC-II expression and reflecting immature myeloid dendritic cells (DC) [14]. Most work has been directed at characterisation of this novel dendritic-like cell called ‘L-DC’ which is produced continuously in these cultures [15, 16]. These findings also led to the hypothesis that hematopoietic stem or progenitor cells were maintained within cultures [17–19]. Stromal lines isolated from spleens of mouse [20–22] have since been shown to support restricted myelopoiesis from overlaid bone marrow progenitors with highly reproducible production of two distinct dendritic-like subsets [22–25]. These two distinct subsets are not developmentally linked and arise through differentiation of distinct progenitors/precursors [23, 24]. The progenitor source of cells produced was identified when highly purified hematopoietic stem cells and progenitors were overlaid above stroma [16, 24, 26]. The production of CD11b^+^CD11c^+^MHC-II^−^CD8α^- ‘^L-DC’ was shown to be continuous and dependent on overlay of early hematopoietic stem cells or multipotential progenitors above the stroma [16]. A second subset of CD11b^+^CD11c^+^MHC-II^lo^CD8α^−^ DC resembling well known conventional (c) DC was found to be transiently produced and dependent on overlay of myeloid progenitors present in mouse bone marrow [16]. L-DC activate CD8^+^ T cells, but not CD4^+^ T cells [23, 24]. The cDC-like population is produced transiently for only 2–4 weeks, consistent with development from myeloid precursors which are not self-renewing. Because these cDC-like cells have limited replicative capacity in stromal co-cultures, their functional capacity has not been fully established [24]. They are here investigated in terms of their similarity with L-DC and with reported subsets of DCregs produced in vitro.

## Results

### Production of dendritic-like cells in stromal co-cultures

**Fig. 1:**
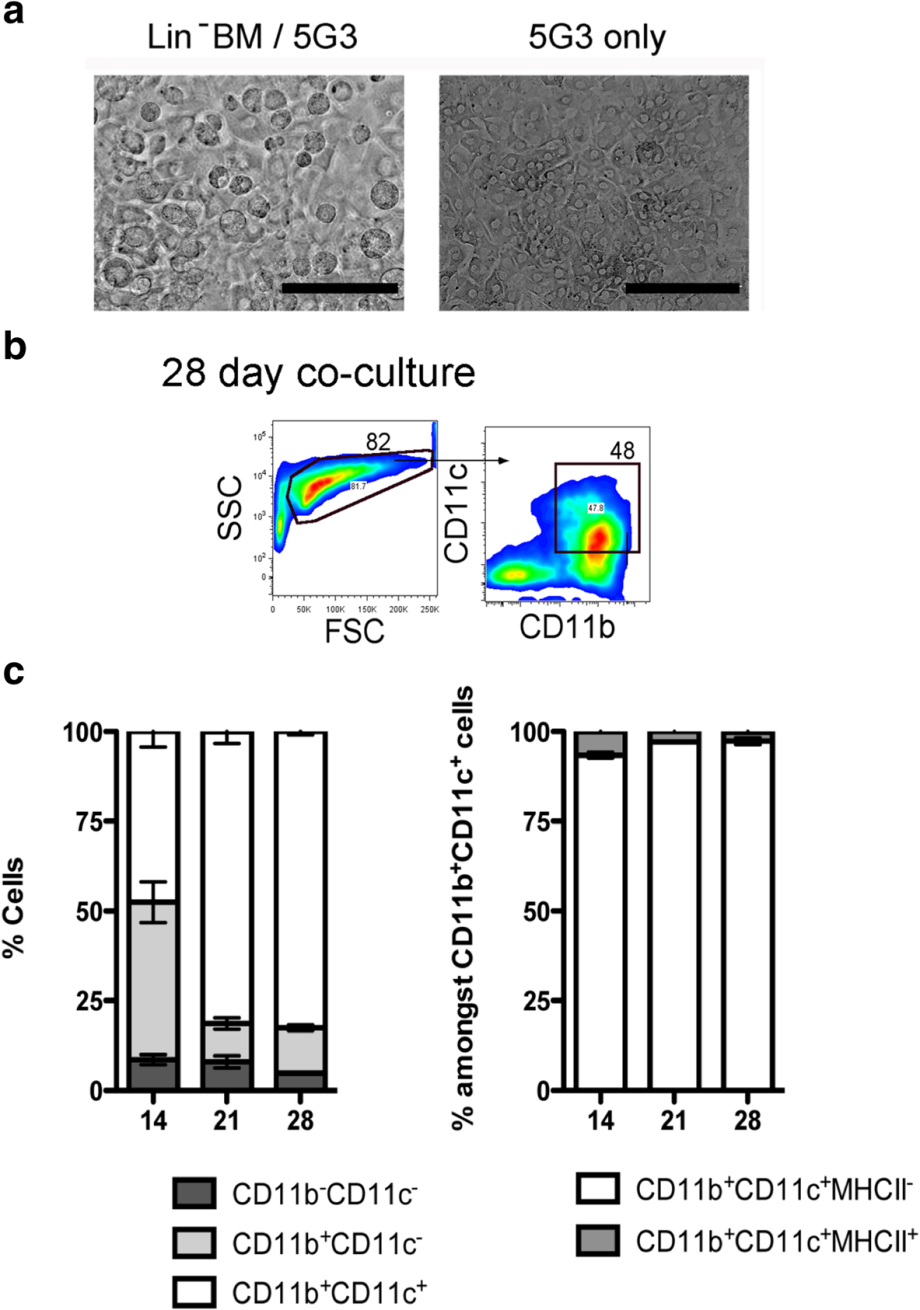
Production of cells in stromal co-cultures. Co-cultures were established by overlay of Lin^−^ bone marrow over 5G3 stroma. (**a**) Hematopoietic cells adhered to stroma were observed under phase contrast microscopy at day 21. Bar represents 100 μm. (**b**) Gating procedure to detect CD11b^+^CD11c^+^ cells is shown for cells produced in co-cultures at 28-days. (**c**) Cell production was assessed at 14, 21 and 28 days using antibody staining and multicolor flow cytometry. Production of the CD11b^−^CD11c^−^, CD11b^+^CD11c^−^, CD11b^+^CD11c^+^, CD11b^+^CD11c^+^MHC-II^−^(L-DC) and CD11b^+^CD11c^+^MHC-II^+^ (cDC-like) subsets was calculated in terms of proportion of each subset amongst the total CD11b^+^ and/or CD11c^+^ population. Graphs show mean ± S.E. for triplicate co-cultures. Cell production in terms of subset size is statistically different (*p* ≤ 0.05) for days 21 and 28 compared with day 14 in terms of increased dendritic cell and reduced myeloid cell production

Co-cultures of 5G3 stroma were established with Lin^−^ bone marrow from C57BL/6 J mice as an overlay to establish hematopoiesis for production of dendritic-like cells. Growth of hematopoietic cells was evident over time (Fig. 1). Non-adherent cells were collected and analysed by antibody staining and flow cytometry at 14, 21 and 28 days to detect the two common dendritic-like subsets previously described [23, 24]. Analysis involved gating PI^−^ live large (FSC^hi^) cells on the basis of CD11b and CD11c expression (Fig. 1B). The CD11b^+^CD11c^+^ subset of dendritic-like cells was then further divided on the basis of MHC-II, CD8α and B220 expression for delineation of CD11b^+^CD11c^+^MHC-II^−^CD8α^−^B220^−^ ‘L-DC’ and CD11b^+^CD11c^+^MHC-II^+^CD8α^−^B220^−^ ‘cDC-like cells’ as described previously [23, 24]. The production of CD11b^+^CD11c^+^ dendritic-like cells stably increased in co-cultures over time, approaching 80% at 21 and 28 days (Fig. 1C). A 40% subset of CD11b^+^CD11c^−^ myeloid cells was observed at 14 days, with a significant decrease in this population to < 15% by 21 days. The CD11b^−^CD11c^−^ progenitor subset was maintained at < 10% over time. L-DC as CD11b^+^CD11c^+^MHC-II^−^ cells were the majority population representing > 90% of CD11b^+^CD11c^+^ cells, with a minor subset (< 8%) of cDC-like as CD11b^+^CD11c^+^MHC-II^+^ cells maintained across 14 to 28 days, indicating two distinct cell types.

**Fig. 2:**
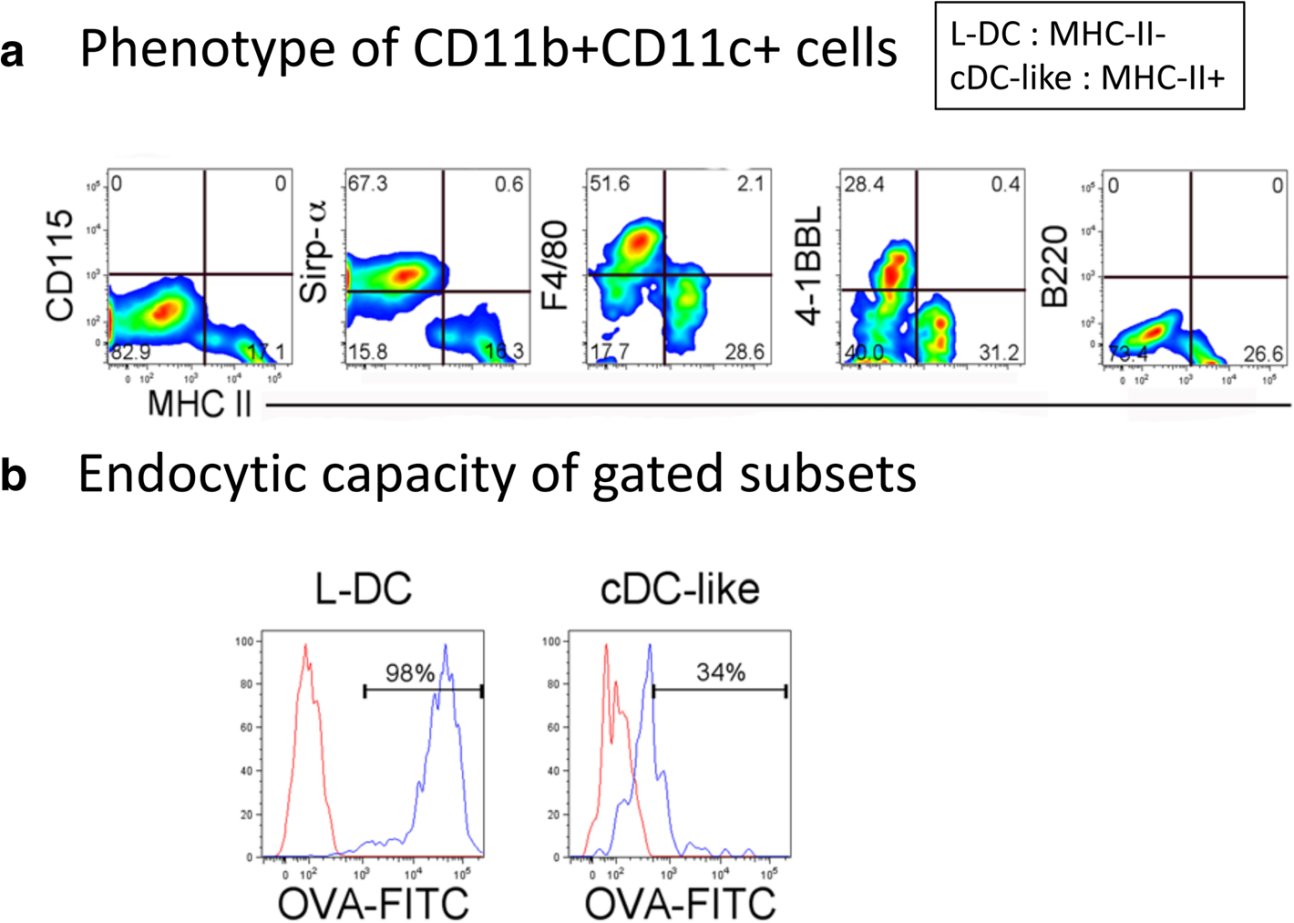
Phenotype of dendritic-like cells produced in co-cultures. Lin^−^ BM from C57BL/6 J mice was overlaid on 5G3 stroma to establish co-cultures and non-adherent cells were collected over time for staining with a range of antibodies for flow cytometric assessment of marker expression. (A) The CD11b^+^CD11c^+^ population of dendritic-like cells produced in 21-day co-cultures was gated for further analysis of marker expression. L-DC and cDC-like cells were distinguished through expression of MHC-II, whereby L-DC are CD11b^+^CD11c^+^MHC-II^−^ cells and cDC-like cells can be distinguished as CD11b^+^CD11c^+^MHC-II^+^ cells. Bivariate analysis of MHC-II expression with CD115, Sirp-α, F4/80, 4-1BBL, B220 and CD8α, served to identify the phenotype of the 2 subsets. (B) Non-adherent cells collected from 21 day co-cultures were incubated with FITC-ovalbumin (OVA) at 37 °C, or at 4 °C as control, to assess capacity of cells to endocytose antigen. At the end of incubation, cells were stained for markers, and L-DC and cDC-like subsets gated on the basis of phenotype as CD11b^+^CD11c^+^MHC-II^−^ and CD11b^+^CD11c^+^MHC-II^+^ cells, respectively. These were then assessed flow cytometrically for endocytosis in terms of % cells taking up FITC-ovalbumin (OVA) (red histogram) compared with controls (blue histogram)

By 21 days, further analysis of cell subset phenotype was possible since clear subsets of L-DC (CD11b^+^CD11c^+^MHC-II^−^) and cDC-like cells (CD11b^+^CD11c^+^MHC-II^+^) can be distinguished. Cells were therefore stained with F4/80, 4-1BBL and Sirp-α to further distinguished the two distinct dendritic-like subsets (Fig. 2). While the cDC-like cells expressed none of these marker, majority subsets of L-DC expressed Sirp-α and F4/80, with a minority subset expressing 4-1BBL (Fig. 2). Lack of CD115 and B220 expression by both DC subsets distinguished these cells from pDC and cDC precursors [27]. The expression of F4/80 has been reported previously on some but not all CD11c^+^ DC produced in spleen stromal cultures [16, 23], and is no longer considered an exclusive marker of monocytes/macrophages. The expression of 4-1BBL by L-DC is consistent with capability of L-DC to induce CD8^+^ T cell proliferation [28]. Sirp-α staining also served to distinguish the two cell types produced in co-cultures. It is a marker expressed by some DC and macrophage subsets but is expressed only by L-DC in co-cultures (Fig. 2A) [29].

To determine the endocytic capacity of L-DC and cDC-like cells produced in co-cultures, cells were collected from co-cultures and incubated with ovalbumin (OVA)-FITC. After 45 min incubation, endocytosis was halted, and cells stained for CD11b, CD11c and MHC-II to identify L-DC and cDC-like subsets, and to determine capacity for uptake of OVA-FITC. L-DC were highly endocytotic cells with 98% taking up OVA-FITC, while only 34% of cDC-like cells were endocytotic (Fig. 2B).

### Dendritic-like subsets differ in capacity to induce T cell responses

**Fig. 3:**
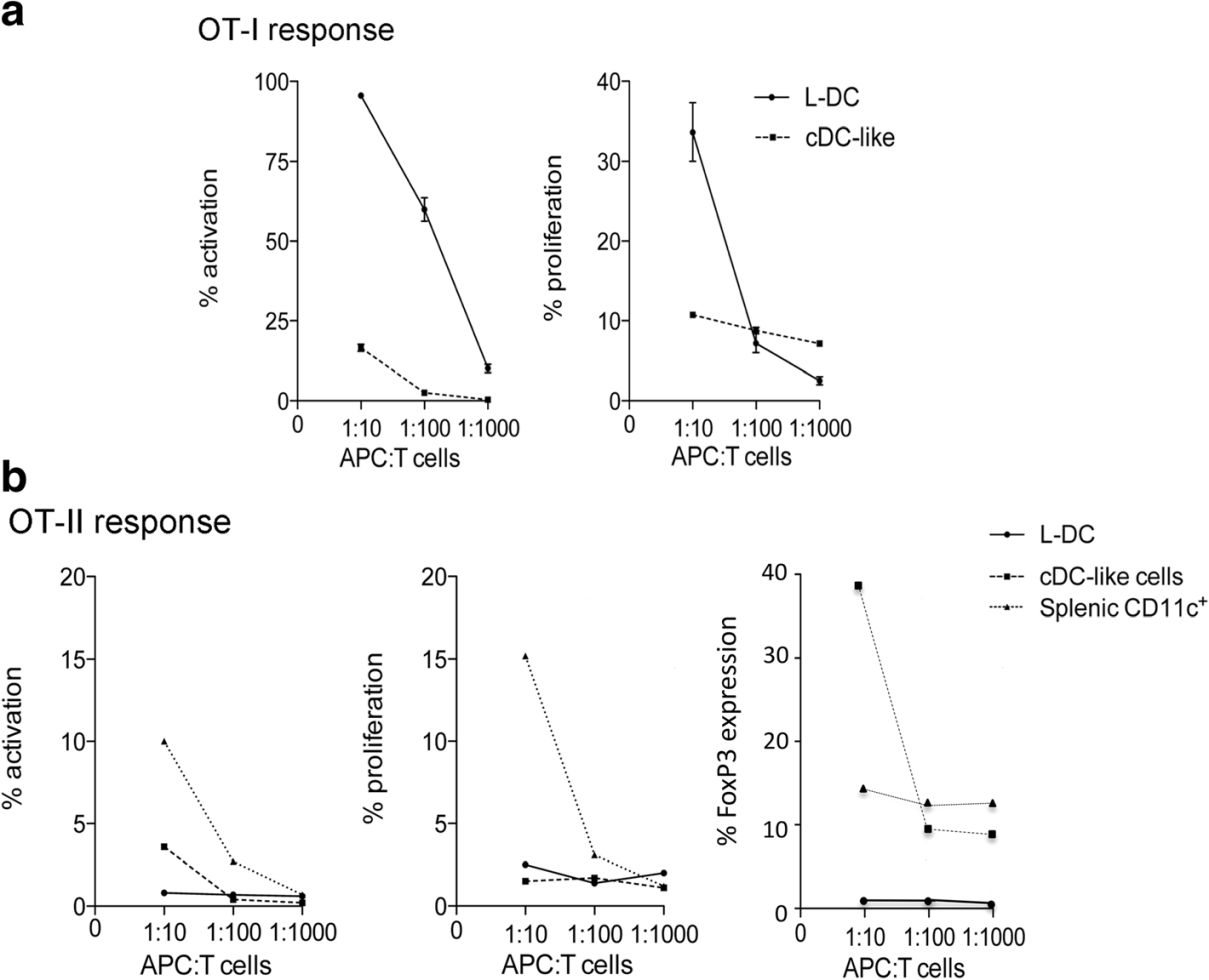
Ability of DC produced in co-cultures to induce T cell responses. Co-cultures were established by overlay of Lin^−^ BM from Act-mOVA mice above 5G3 stroma and non-adherent cells collected after 21 days. Cells were stained for CD11b, CD11c and MHC-II expression and subsets of L-DC and cDC-like cells sorted and then incubated with CD8^+^ T cells purified from OT-I TCR-tg mice specific for OVA_257–264_/H-2K^b^ or OT-II TCR-tg mice specific for OVA_323–339_/H-2IA^b^ at APC:T cell ratios of 1:10, 1:100 and 1:1000. Controls included T cells only with no APC. Control APC included CD11c^+^ DC from spleen. (**a**) Cells for analysis of OT-I responses were collected from cultures and gated as live (PI^−^) Thy1.2^+^Vα2^+^CD8^+^ T cells. T cell activation was measured at 24 h in terms of % cells expressing CD69. T cell proliferation was measured after 4 days in terms of a reduction in CFSE staining. Data represent mean ± S.E. of three replicate co-cultures. L-DC gave statistically greater T cell activation across all three APC:T cell ratios, while statistically greater T cell proliferation was noted only at an APC:T cell ratio of 10:1 (*p* ≤ 0.05). (**b**) Cells for analysis of OT-II responses were collected from cultures and T cells gated as live (PI^−^) Thy1.2^+^Vα2^+^CD4^+^ cells. T cell activation was measured at 24 h as % cells expressing CD69. T cell proliferation was measured at 4 days in terms of reduction in CFSE staining. Production of regulatory T cells was assessed through intracellular Foxp3 expression in CD4^+^ T cells analysed after 4 days by flow cytometry

In order to assess capacity of in vitro produced dendritic-like cells for T cell activation, co-cultures were established using Lin^−^ BM from C57BL/6 J Act-mOVA mice over 5G3 stroma. After 21 days, L-DC and cDC-like cells were sorted, and these two subsets compared for ability to present exogenous antigen to CD8^+^ T cells purified from OT-I TCR-tg mice. L-DC clearly activated CD8^+^ OT-I cells, evident by upregulation of CD69 expression on gated CD8^+^Vα2^+^Thy1.2^+^ T cells after 24 h (Fig. 3A). cDC-like cells were much less capable of activating CD8^+^ T cells. L-DC showed high capacity for inducing both antigen-specific T cell activation and proliferation of CD8^+^ OT-I T cells after 4 days, while cDC-like cells showed only a weak response (Fig. 3A).

**Fig. 4:**
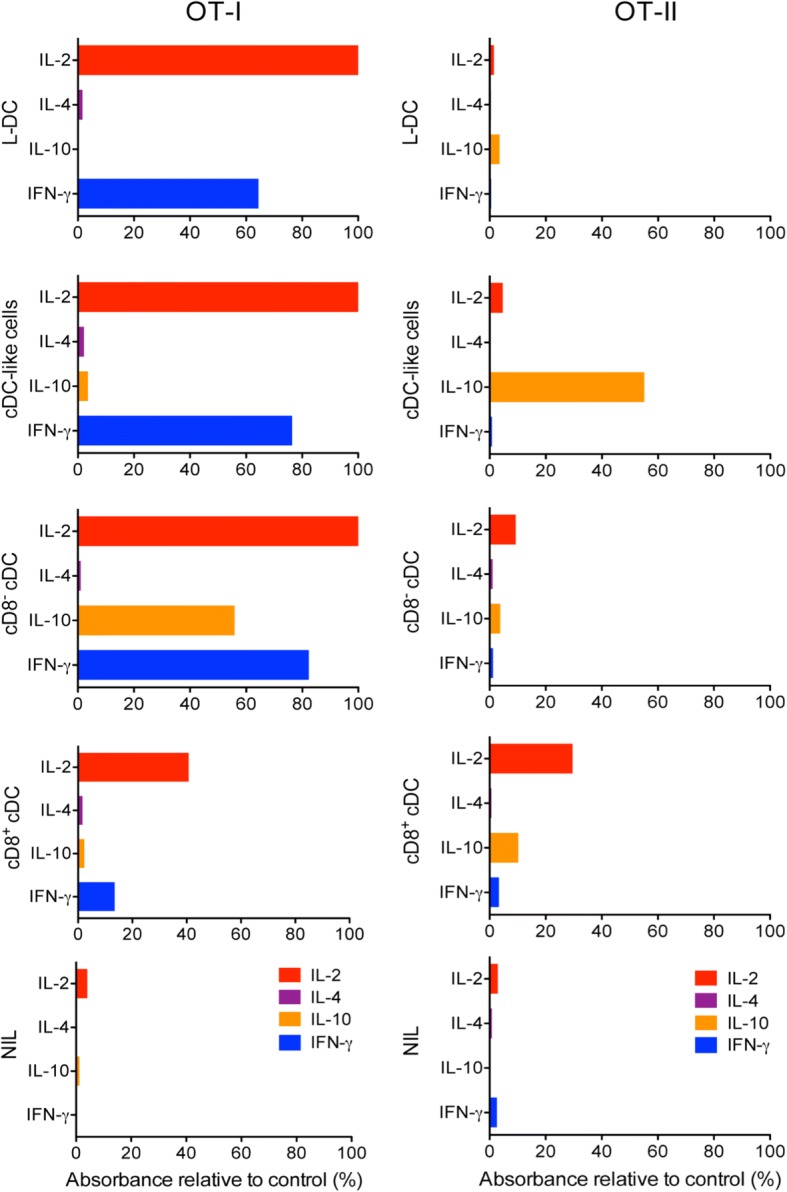
Cytokine release upon DC activation of antigen-specific T cells. The release of IL-2, IL-4, IL-10 and IFN-γ by OT-I and OT-II T cells following stimulation with different APC subsets was investigated. L-DC and cDC-like subsets were sorted from 21-day co-cultures established with Lin^−^ BM from Act-mOVA mice above 5G3 stroma. Control CD8^+^ cDC and CD8^−^ cDC subsets were sorted from Act-mOVA spleen. These four APC subsets, and a NIL control, were incubated with column purified CD8^+^ OT-I T cells and CD4^+^ OT-II T cells from spleen for 4 days. The supernatant of co-cultures was collected, tested for the presence of cytokines using an Elisa array kit (Qiagen), and production calculated as A_450_ of sample relative to A_450_ of a standard provided with the kit

Further evidence of the ability of both L-DC and cDC-like cells to activate OT-I T cells was indicated by the production of IL-2 and IFN-γ in the 4-day supernatant of OT-I CD8^+^ T cells co-cultured with L-DC and cDC-like cells (Fig. 4). While cDC-like cells showed only weak activation of CD8^+^ T cells within 24 h (Fig. 3), they still induced levels of IL-2 and IFN-γ comparable with T cells activated with L-DC, and with T cells activated by the control APC subsets of CD8^+^ cDC and CD8^−^ cDC sorted from normal adult spleen (Fig. 4). In these experiments we also showed that CD8^−^ cDC were stronger activators of OT-I T cells than CD8^+^ cDC in terms of IL-2 and IFN-γ produced (Fig. 4). The production of IL-10 by CD8^−^ cDC activating OT-I T cells could reflect contaminating regulatory DC within this sorted population (Fig. 4).

### In vitro generated cDC-like cells but not L-DC reflect DCregs

For assessment of capacity to induce CD4 T cell responses, sorted L-DC and cDC-like cells, along with freshly isolated splenic CD11c^+^ cells as controls, were cultured with CD4^+^ T cells purified from OT-II mice. L-DC were unable to activate CD4^+^ T cells measured through upregulation of CD69 expression within 24 h, or to induce their proliferation after 4 days (Fig. 3B). In contrast, cDC-like cells induced weak CD4^+^ T cell activation (3.6% of cells) through upregulation of CD69 expression after 24 h, while splenic CD11c^+^ DC showed strong activation (10.1% of cells) (Fig. 3B). Neither L-DC nor cDC-like cells were able to induce CD4^+^ T cell proliferation while splenic CD11c^+^ DC were, however, strong inducers of CD4^+^ T cell proliferation.

The inability to activate CD4^+^ T cells was also shown through analysis of cytokine production by OT-II CD4^+^ cells stimulated for 4 days with sorted L-DC, cDC-like cells, or control CD8^+^ cDC and CD8^−^ cDC (Fig. 4). Neither L-DC nor cDC-like cells induced production of IL-2, IL-4 or IFN-γ, consistent with inability to activate or induce proliferation of OT-II CD4^+^ T cells (Fig. 4). However, cDC-like cells produced high levels of IL-10 in OT-II CD4^+^ T cells which is commonly associated with regulatory or immunosuppressive DC, rather than DC which activate T cell responses [4]. CD8^+^ cDC also produced a low level of IL-10, although at 5-fold lower level than cDC-like cells. CD8^+^ cDC and CD8^−^ cDC sorted from spleen induced production of IL-2 but not IL-4 or IFN-γ after 4 days of culture with OT-II CD4^+^ T cells (Fig. 4).

The possibility that cDC-like cells are DCregs was investigated in terms of their capacity to induce differentiation and activation of CD4^+^ OT-II T cells to Tregs. This involves expression of FoxP3 in T cells. L-DC and cDC-like cells were sorted from 21-day co-cultures of Lin^−^ bone marrow over 5G3 and compared for ability to induce FoxP3 expression in sorted CD4^+^ OT-II T cells along with control CD11c^+^ spleen cells. This involved flow cytometric assessment of FoxP3 expression through intracellular staining of gated CD4^+^ T cells. The response due to cDC-like cells showed 39% of CD4^+^ T cells expressing FoxP3 at an APC:T cell ratio of 1:10, compared with almost none (0.8%) for L-DC, with a partial response (14%) for splenic CD11c^+^ DC (Fig. 3B). This result supported the finding of IL-10 production by CD4^+^ OT-II T cells following incubation with cDC-like cells, but not with L-DC (Fig. 4). These two distinct results are consistent with the hypothesis that cDC-like cells are DCregs.

### Differential gene expression by L-DC and cDC-like cells

Expression of 84 genes identified as markers of murine DC and APC was assessed for the L-DC and cDC-like subsets sorted out of 28-day co-cultures of Lin^−^ bone marrow cells over 5G3 stroma. RNA was prepared for realtime PCR involving the RT^2^ Profiler PCR array produced by SABiosciences. Results were averaged from two separate sorting experiments. Fifty out of 84 genes were differentially expressed by ≥ 2-fold between the two subsets (Table 1). The differential gene expression profiles are consistent with two distinct dendritic-like cell types.

**Table 1:** Genes upregulated in L-DC or cDC-like cells

Gene	L-DC/cDC-like	Gene	cDC-like/L-DC
*Ccl20*	7.30	*Fcer1a*	14.44
*Il12a*	5.53	*Ccl8*	7.96
*Cd8a*	5.38	*Ccl7*	5.78
*Cxcl12*	4.99	*Ccr3*	5.43
*Ccl19*	4.88	*Fcgrt*	5.25
*Ifng*	4.31	*Fcer1g*	5.21
*Cxcl10*	4.16	*Inhba*	4.83
*Vcl*	3.86	*Cd2*	3.92
*Cd1d2*	3.75	*Cd36*	3.79
*Relb*	3.50	*Cxcl2*	3.64
*Il8ra*	3.48	*Tlr2*	3.51
*Adamdec1*	3.41	*Ccl2*	3.42
*Il2*	3.31	*Mif*	3.42
*Ifit3*	3.29	*Ccl12*	3.39
*Erbb2*	3.11	*Ccr5*	3.37
*Icam2*	2.99	*Csf1r*	2.72
*Ccl5*	2.84	*Ccr1*	2.62
*Ccr2*	2.58	*Cd33*	2.55
*Icam1*	2.39	*Itgb2*	2.22
*Ccl11*	2.34	*Il16*	2.19
*Fcer2a*	2.33	*Cebpa*	2.18
*Cd80*	2.31	*Tnf*	2.16
*Cd4*	2.25	*Cdc42*	2.00
*Cd40*	2.23		
*Trap1*	2.14		
*Il12b*	2.11		
*Il6*	2.11		

Realtime PCR was performed using an RT^2^ Profiler PCR Array (SABiosciences) to measure the relative expression of genes common to murine dendritic and antigen presenting cells by sorted populations of L-DC and cDC-like cells isolated from 28-day co-cultures. Genes upregulated ≥ 2-fold in L-DC or cDC-like cells are listed

The majority of genes upregulated in L-DC encode markers of mature, immunostimulatory DC. These include *Il12a* (interleukin 12α), *Il12b* (interleukin 12β), *Ifng* (interferon γ), *Il6* (interleukin 6) and *Il2* (interleukin 2), as well as genes encoding cell surface markers of DC including *Cd80, Cd4, CD8a, CD40* and *Adamdec1* [30, 31]. Cells also express *Relb* which is expressed by activated DC [32], as well as *Vcl* (vinculin) important for antigen uptake [33], and *Cd1d2* encodes an MHC-like antigen presenting molecule for activation of Natural Killer T cells [34]. These cells also show upregulation of genes for the proinflammatory factor *Ccl20* (MIP-3A), and chronic inflammatory factors *Ccl5* and *Ccl11*.

Genes upregulated by cDC-like cells reflect a myeloid dendritic cell type with potential immunosuppressive capacity in line with classification of cells as DCregs. The most highly upregulated genes included *Fcer1a* which encodes an Fc receptor for IgE binding which could trigger DC to activate T cells in response to allergen exposure. The cells also express *Tlr2* encoding toll-like receptor 2, which makes them sensitive to pathogen activation. However, several other upregulated genes suggest capacity of cDC-like cells to be involved in suppressive responses. Expression of *Inhba* encoding activin-βΑ a member of the TGF-β family, is consistent with capacity to induce formation of T regs [35]. Expression of *Itgb2* which encodes β2 integrin can lead to suppression of Toll-like receptor stimulation [36]. Several other genes encoding chemokines associated with inflammatory responses associated with autoimmunity were found to be upregulated. These included *Ccl8, Ccl7, Ccl2, Ccl12* and *Tnf.* Myeloid cell characteristics of cDC-like cells are indicated by expression of *Cebpa,* a transcription factor for DC development from progenitors [37], *Cd33* which encodes a marker of myeloid and also myeloid suppressor cells [38], *Csfr1* encoding MCSFR a common marker of myeloid lineage monocytes/macrophages, *Cd36* which encodes a phagocytic receptor [39]*, Mif* which encodes a suppressive factor involved in phagocytosis, recognition and engulfment of antigen [40]*,* and *Ccr5* which encodes a chemokine receptor present on DC entering inflammatory sites [41].

### M-CSF directs the development of DCregs in stromal co-cultures

Previously it was shown that the production of L-DC in co-cultures established above 5G3 splenic stroma could be completely inhibited if bone marrow progenitors were plated above a Transwell membrane to prevent cell-cell contact with the stromal cell monolayer [24]. These co-cultures generated instead an enriched population of cells highly enriched for cDC-like cells. Previous studies from this lab also identified macrophage colony stimulating factor (MCSF) as an important factor for the generation of cDC-like cells [16], and this is produced at high levels by splenic stromal lines [42]. In contrast, L-DC production was entirely dependent on stromal cell interaction [16]. Data in Table 1 has confirmed nearly 3-fold higher expression of *Csfr1* in cDC-like cells compared with L-DC after 28 days of co-culture, despite the fact that cells have lost cell surface receptor expression for CD115 (CSFR1/MCSFR) (Fig. 2).

**Fig. 5:**
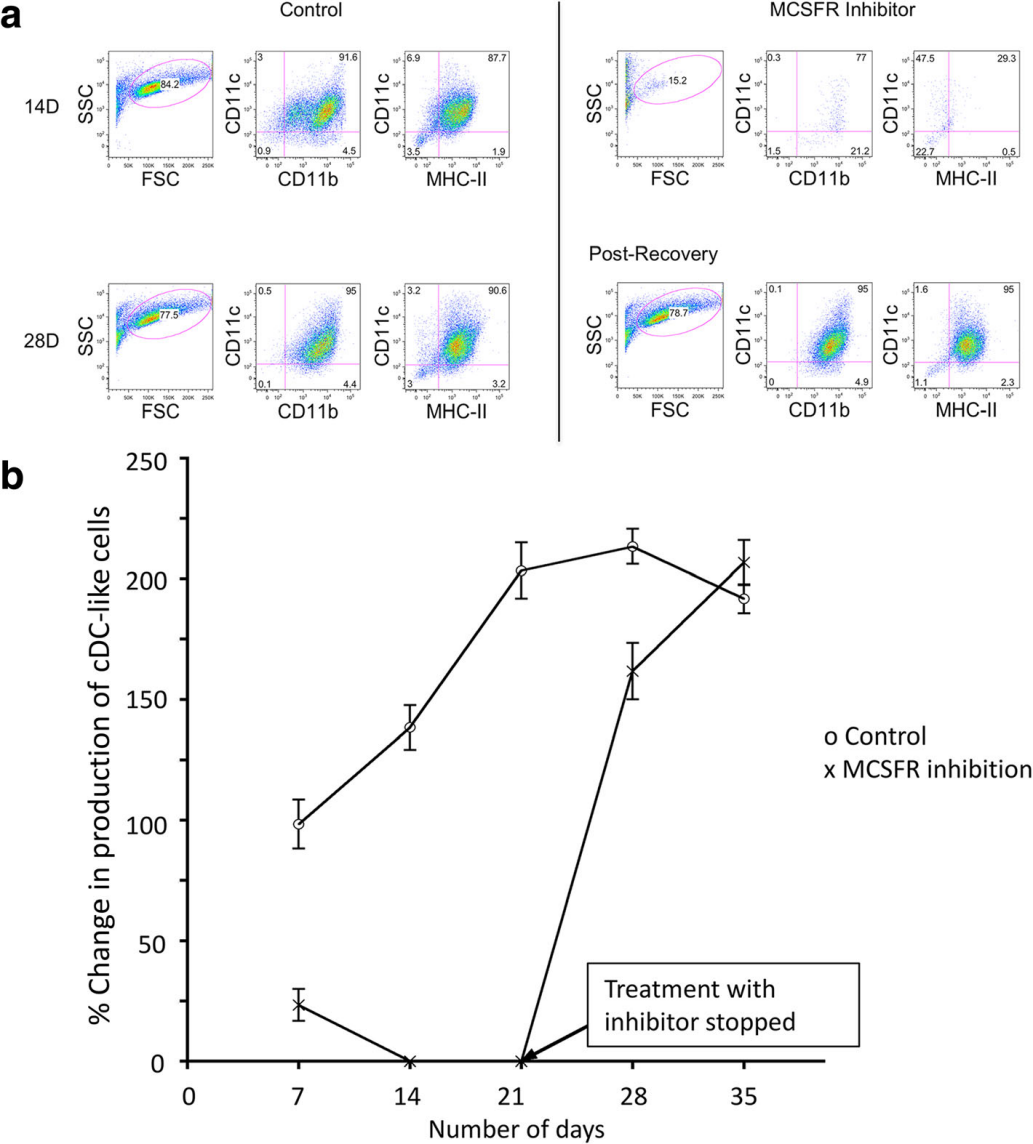
MCSF-dependency of DCreg development. Co-cultures were established by overlay of Lin^−^ BM from C57BL/6J mice above a Transwell membrane to prevent contact of between hematopoietic progenitors and 5G3 stroma. Three replicates cultures were treated with the M-CSFR inhibitor GW2580 (10 mM), or left untreated (controls), with inhibitor replenished every 3 days at medium change. Treatment continued for 21 days, followed by a period of 14 days in the absence of inhibitor to assess recovery. Cell production was analysed flow cytometrically every 7 days through antibody staining to detect CD11b, CD11c and MHC-II expression and to delineate CD11c^+^CD11b^+^MHC-II^+^ cDC-like cells. (**a**) Gating procedure for identification of cDC-like cells produced in 14-day Transwell co-cultures in the presence of inhibitor and in 28-day co-cultures after inhibitor has been washed out. Controls contained no inhibitor. (**b**) Production of DCregs in Transwell co-cultures expressed in terms of number of cDC-like cells produced over time relative to cell number produced in 7-day control cultures. Data represent mean ± SE of three replicate co-cultures. Addition of GW2580 significantly inhibited cell production relative to control at each 7-day interval out to 28 days (*p* ≤ 0.05)

Co-cultures established with Lin^−^ bone marrow progenitors seeded above a Transwell membrane preventing contact with stroma, were highly productive of cDC-like cells with no L-DC production (Fig. 5). The production of cDC-like cells doubled across 7 to 21 days and maintained this level of production over 35 days. MCSF dependency for cell production under non-contact growth conditions was confirmed through addition of the specific MCSFR inhibitor GW2580. This was replenished every 3 days at medium change. After 14 days of culture in the presence of inhibitor, production of cDC-like CD11c^+^CD11b^+^MHCII^+^ cells had ceased (Fig. 5A and B). Following 21 days of treatment, cultures were then returned to normal medium, and the production of cells resumed, reaching equivalence with control cultures by 35 days (Fig. 5B). Post recovery cells at 28 days showed the same phenotypic characteristics as cells produced in control cultures (Fig. 5A). This result confirms that inhibition of MCSFR served only to prevent the production of cells and did not destroy the progenitor/precursor population over 21 days of treatment.

## Discussion

This study confirms the work of others by demonstrating the ability to produce DCregs from bone marrow progenitors cultured above spleen stroma [9, 11–13]. It goes further, however, in that it differentiates the production of DCregs from the novel L-DC subset reported previously by this lab. L-DC reflect a novel dendritic-like cell type produced continuously in spleen stromal co-cultures overlaid with bone marrow progenitors [14, 15]. An in vivo equivalent L-DC subset was recently described in both murine [43] and human spleen [44], which gives physiological relevance to the hematopoietic process giving rise to L-DC in vitro. L-DC are quite distinct, both phenotypically and functionally, from all known myeloid and dendritic cell types in spleen, including DCregs [43, 45]. A series of studies have also determined that L-DC develop independently of MCSF, FLT3L, GMCSF, and also without BATF3 expression which drives cDC and pDC development [45], and also c-MYB, a transcription factor important in the development of bone marrow-derived myeloid cells [16, 46]. All work to date points to L-DC arising from hematopoietic progenitors endogenous to spleen which may have been laid down in spleen during embryogenesis.

This study investigates the cDC-like population of cells produced transiently in spleen stromal co-cultures seeded with bone marrow and demonstrates the unique importance of MCSF in their development. While other soluble factors produced by 5G3 may direct cDC-like cell development, these are not deterministic. In our hands, production of cDC-like cells reflecting DCregs depends on the prevention of L-DC production in co-cultures established from Lin^−^ bone marrow by using a Transwell membrane. The latter method introduces a simple procedure with application in production of autologous DCregs for therapeutic use.

An important role for MCSF has been confirmed in the production of cDC-like cells in co-cultures. While neither the L-DC or cDC-like subsets produced in Lin^−^ bone marrow co-cultures over 5G3 stroma expressed CD115 (MCSFR) after 28 days of co-culture (Fig. 2), this result does not preclude a role for MCSF in the early development of cells from myeloid progenitors/precursors or monocytes in co-cultures. Since MCSF is important in cell development it is likely that DCregs develop from a CD115^+^ precursor present in bone marrow which differentiates in response to MCSF produced by stroma. This is consistent with former work showing that progenitors of the cDC-like population were present within each of the defined subsets of Lin^−^Sca1^−^ckit^hi^Flt3^+^CD115^+^CX3CR1^−^ myeloid progenitors [47, 48], the Lin^−^Sca1^−^ckit^hi^Flt3^+^CD115^+^CX3CR1^+^ myeloid/dendritic progenitor subset [49], and the Lin^−^Sca1^−^ckit^lo^Flt3^+^CD115^+^ common dendritic progenitor subset [50]. There is still ambiguity about the delineation of these three progenitor subsets, and evidence that they reflect overlapping subsets [49], a finding consistent with CD115 expression by precursors of DCregs. Furthermore, dependency on MCSF for development also serves to distinguish cDC-like cells from CD8^−^ cDC and confirms that they were not immediate precursors of cDC or pDC [27, 51].

In light of evidence for a role of M-CSF in the development of cDC-like cells reflective of DCregs, the question of whether co-cultures over spleen stroma produce myeloid lineage cells or macrophages needs to be addressed. Over many studies we have shown that co-cultures established with Lin^−^ bone marrow produce a low yield of CD11b^+^CD11c^−^MHC-II^−^ cells which we have classified as ‘myeloid’. These cells could be either myeloid progenitors/precursors or monocytes/macrophages. Most recently, the studies of Periasamy et al. [16] identify a small population of these cells after 14 days of co-culture which are almost gone by 21 days. Petvises & O’Neill [24] also shows evidence of a small population produced in co-cultures of Lin^−^ bone marrow over 5G3 stroma. They are only detectable in co-cultures using Lin^−^ bone marrow as a source of progenitors and are not produced in co-cultures established with purified HSPC [16]. Hence the myeloid subset may arise from myeloid progenitors/precursors present in Lin^−^ bone marrow. Since this population is only enriched (95%) through magnetic bead depletion, it is possible that it also contains some more mature myeloid cells as contaminants. Because of the small size of this population, myeloid cell production has not been investigated further. Since these cells are transiently produced over just several weeks, they have not been of interest in light of the evidence that L-DC are produced continuously, and cDC-like cells are produced in higher numbers.

Previous evidence showed that the addition of a Transwell to co-cultures of Lin^−^ bone marrow co-cultured with 5G3 stroma inhibited L-DC production and gave a marked increase in cDC-like cell production [24]. These results suggested that L-DC production was dependent on stromal cell contact, while cDC-like production was not. Further studies performed in the absence of a Transwell, showed that addition of the MCSFR inhibitor GW2580 affected production of dendritic-like cells which was manifest as a loss of cDC-like cells (CD11b^+^CD11c^+^MHC-II^+^) with an increase in L-DC (CD11b^+^CD11c^+^MHC-II^−^) production [16]. This suggested that the production of the two cell types was distinct and in dynamic equilibrium, with the L-DC subset dependent on stromal contact and the cDC-like subset dependent on M-CSF for development. These Lin^−^ bone marrow co-cultures showed very low production of CD11b^+^CD11c^−^ myeloid cells, which was not inhibited by GW2580 [16]. Figure 5 now shows Lin^−^ bone marrow co-cultures established in the presence of a Transwell, where many more cDC-like cells than L-DC are produced. This is distinct from results shown in Fig. 2, where similar co-cultures established in the absence of a Transwell, produce more L-DC than cDC-like cells. The data in Fig. 5 then shows clearly that production of cDC-like cells above the Transwell can be very effectively inhibited by GW2580, suggesting dependency on M-CSF for production.

While little is known about the development and lineage origin of DCregs, it is clear that the population is heterogeneous both in the mouse and human models. DCregs are known to retain the ability to present antigen for activation of T cells, evident through their ability to activate CD8^+^ T cells [6]. However, DCregs downregulate their capacity to activate CD4^+^ T cells through downregulation of IL-12 production and reduced expression of markers like MHC-II, CD80, CD86 and CD40. At the same time, they also increase their immunosuppressive capacity through expression of inhibitory molecules like CD95L, IDO and PDL1, and production of inhibitory cytokines like TGF-β and IL-10 [4, 6]. DCregs produced here show functional evidence of their immunosuppressive capacity through secretion of IL-10 upon co-culture with antigen-specific CD4^+^ T cells. In comparison with L-DC, they also show upregulation of genes reflective of myeloid dendritic cells which have inflammatory and immunosuppressive characteristics rather than activating capacity.

Currently few markers are available which are delineating for DCreg subsets, and discovery has been impeded by the inability to isolate large numbers of these cells for characterization. The development and role of DCregs is not well understood, and more than one pathway for development is apparent. The availability of reproducible tissue culture systems for production of cells should allow transcriptome analysis and the delineation of transcription factors and gene pathways driving cell development. DCregs represent an important therapeutic tool for the induction of Tregs which can control autoimmunity, inflammation, and unwanted T cells, for example during tissue graft rejection.

## Conclusion

A co-culture system of hematopoietic progenitors over splenic stroma has been shown to produce two distinct DC subsets. Since similar culture systems have been shown to yield DCregs, the two APC subsets were compared in terms of phenotype and function in T cell activation. Here the cDC-like population produced in co-cultures of lineage-depleted (Lin^−^) bone marrow over the 5G3 murine spleen stromal line has been shown to resemble DCregs. Conditions which support specific M-CSF-dependent production of these cells in the absence of L-DC have been defined offering a means for production of enriched populations of these elusive cells, perhaps for specific therapeutic use.

## Methods

### Animals

Specific pathogen-free female C57BL/6.Tg (TcraTcrb)1100Mjb (OT-I), C57BL/6.SJL/J.OT-II.CD45.1 (OT-II), C57BL/6.Tg (CAG-OVA) 916Jen:WehiAnu (Act-mOVA) and C57BL/6J mice were bred at the John Curtin School of Medical Research (JCSMR) (Canberra, Australia). Protocols covering housing, handling and experimentation of animals were approved by the Animal Experimentation Ethics Committee (Australian National University, Canberra, Australia). Animals were sacrificed by cervical dislocation.

### Antibodies

Fluorochrome-conjugated antibodies specific for CD4 (GK1.5), Thy1.2 (30-H12), CD69 (H12F3), B220 (RA3-6B2), MHC-II (AF6–120.1), F4/80 (C1: A3–1), CD8α (53–6.7), Sirp-α (P84), 4-1BBL (TKS-1), streptavidin-PE-Cy7, streptavidin-PE and streptavidin-FITC were obtained from Biolegend. Fluorochrome-conjugated antibodies specific for CD11c (N418), CD11b (M1/70), CD115 (AFS98) and streptavidin-APC-Cy7 were obtained from eBiosciences (San Diego, CA, USA) or Biolegend (San Gabriel, CA, USA). Isotype control antibodies including Rat IgG_2a_-FITC (R35–95), Rat IgG_2b_-PE (RTK4530), Rat IgG_2b_-PE-Cy7 (eB149/10H5), Mouse IgG_2a_-biotin (eBM2a) and Hamster IgG-APC (eBio299Arm) were obtained from eBioscience.

### Preparation of bone marrow cells

This procedure has been described in detail previously [16]. Briefly, medium (DMEM: 5 ml) was used to flush bone marrow from femurs. Cells were centrifuged and resuspended in red blood cell lysis buffer (140 mM NH_4_CL, 17 mM Tris Base [pH 7.5]) for removal of red cells. Lin^−^ bone marrow was prepared using a lineage depletion kit (Miltenyi Biotech, Gladbach, Germany) containing biotinylated antibodies specific for all hematopoietic lineages (7–4, CD5, CD11b, CD45R, Ly6G/C and Ter119) to which was added antibody specific for CD11c (HL3: Becton Dickinson Pharmingen, San Diego, CA, USA) to deplete DC. Anti-biotin microbeads in MS or LS columns (Miltenyi Biotech) were used to purify Lin^−^ bone marrow cells.

### Establishment of 5G3 co-cultures

Cells were cultured as described previously [16] in Dulbecco’s modified Eagles Medium (DMEM) supplemented with 4 g/L D-glucose, 6 mg/L folic acid, 36 mg/L L-asparagine, 116 mg/L L-arginine, to which was added 10% fetal calf serum, 10 mM HEPES, 2 mM L-glutamine, 100 U/L penicillin, 100μg/L streptomycin, and 5 × 10^−5^M 2-mercaptoethanol (sDMEM). The stromal cell line 5G3 [20–22] was passaged every 3–4 days by scraping and transfer of non-adherent cells to a new flask. Cells were maintained in 5% CO_2_ in 95% humidity at 37 °C. For establishment of co-cultures, Lin^−^ bone marrow cells (10^4–5^ cells/ml) were overlaid on to near-confluent 5G3 stroma in replicate 25cm^2^ flasks as described previously [22–24]. Medium change was performed every 3–4 days by replacement of 2.5 ml medium with 2.5 ml sDMEM. This yielded a 5- to 10-fold increase in cell number. Non-adherent cells were collected at days 14, 21 and 28 for analysis of subsets produced. Transwell experiments were achieved by overlay of 10^4–5^ cells/ml Lin^−^ bone marrow cells on to Transwell membranes above 5G3 stroma. For inhibition studies, 10^4–5^ cells/ml Lin^−^ bone marrow cells from C57BL/6 J mice were overlaid on to 5G3 stroma in the presence and absence of the MCSFR inhibitor GW2580 used at 10 nM (BioVision, CA, USA).

### Analysis of cells produced in co-cultures

Non-adherent cells collected from co-cultures at each time point were stained with antibodies specific for myeloid and dendritic-like cells. As described previously [16, 24, 25], 10^5–6^ cells were incubated with CD16/32 antibody (Clone 93; eBioscience) for 15 min to block surface Fc receptors. Cells were then washed with DMEM/1%FCS/0.1%NaN_3_ (FACS buffer) and stained with primary antibodies specific for CD8α, B220, CD115, F4/80, 4-1BBL, CD11c, CD11b, MHC-II and Sirp-α for 20 min on ice. Secondary fluorochrome conjugates were added after a washing step and incubated for a further 20 min on ice. Cells were then washed twice and resuspended in FACS buffer for flow cytometric analysis. Cells were stained with propidium iodide (PI) (1 μg/ml) for live cell discrimination in order to gate PI^−^ live cells. FSC versus SSC plots were used to gate large cells for marker analysis. Cell acquisition involved use of a LSR II flow cytometer (Becton Dickinson), and between 5 × 10^4^ and 1 × 10^6^ events were collected for each sample. Gates were set to delineate cell subsets using isotype control antibodies and ‘fluorescence minus one’ (FMO) controls. Numbers on gates reflect % positive cells. Cell subset analysis was performed using BD FACSDiva Software (Becton Dickinson) and FlowJo Software (Tristar; Phoenix, Arizona, USA).

### Endocytosis

Capacity of cells to take up antigen was assessed by in vitro measurement of endocytosis as described previously [23]. Cells were washed twice with DMEM and placed on ice for 10 min. FITC-conjugated ovalbumin (OVA-FITC) (Molecular Probes, Eugene, OR, USA) (2 μg/ml) was added and cells incubated at 37 °C for 45 min, or at 4 °C as a control. The addition of 100 μl chilled PBS/0.1%NaN_3_ was used to halt the reaction. Cells were washed thrice by centrifugation at 300 g and 4 °C for 5 min, supernatant discarded completely, and cells resuspended in 70 μl of FACS buffer for flow cytometric analysis.

### Purification of T cell subsets

This procedure was described previously [24]. Briefly, spleen cells were resuspended in medium containing monoclonal antibodies specific for APC including macrophages, B cells and DC. The antibody cocktail contained anti-CD11b (clone: M1/70), anti-B220 (clone: RA3-6B3) and anti-IA^b/k^ (clone: TIB120) per 10^7^ cells. For depletion of CD4^+^ or CD8^+^ T cells, either anti-CD4 (GK1.5) or anti-CD8 (53–6.7) was included in the cocktail. Cells were incubated with antibodies on ice for 25 min followed by washing twice with buffer. Supernatant was discarded completely, and cells then incubated with sheep anti-rat Ig Dynabeads® (Invitrogen Dynal: AS, Oslo, Norway) (50 μl beads/10^7^cells) at 4 °C for 25 min with rotation. Sheep anti-rat Ig Dynabeads® were washed twice with buffer prior to use. Following incubation, cells were placed in a Dynal® magnetic particle separator for 2 min. Supernatant containing unbound T cells was transferred into a new tube. Enriched T cell subsets were washed, and cell number determined before use.

### T cell activation and proliferation

The procedures used here were adapted from previously published protocols [24]. Splenic CD8^+^ T cells purified from OT-I T cell receptor (Vα2)-transgenic (TCR-tg) mice specific for ovalbumin (OVA_257–264_/H-2K^b^) were employed to determine the antigen presentation capacity of DC. APC were isolated as L-DC sorted as CD11b^+^CD11c^+^MHC-II^−^ and cDC-like cells sorted as CD11b^+^CD11c^+^MHC-II^+^ from 21-day co-cultures of Lin^−^ BM from Act-mOVA mice cultured above 5G3 stroma. Splenic CD11c^+^ cells from Act-mOVA mice were prepared as control DC. All DC subsets were plated in 96-well plates in sDMEM together with purified splenic OT-I CD8^+^ T. APC and T cell were plated at ratios of 1:10, 1:100, and 1:1000, in a total volume of 200 μl. Cells were incubated at 37 °C, 5% CO_2_ in air and 95% humidity for 24 h, and then CD8^+^T cells assessed for activation through expression of CD69. For assessment of CD8^+^ T cell proliferation, similar experiments were conducted using CFSE-labeled CD8^+^ T cells. Cells were cultured for 4 days and then stained with antibody to CD8, Vα2 and Thy1.2, and proliferation determined in terms of CFSE dilution by flow cytometry. Supernatant was collected and frozen at − 20 °C for determination of cytokine released.

The ability of APC subsets to present antigen to CD4^+^ T cells was demonstrated through ability of antigen-pulsed cells to induce proliferation of purified CD4^+^ T cells isolated from OT-II TCR (Vα2)-tg mice specific for ovalbumin (OVA_323–339_/H-2IA^b^). Procedures for isolation of DC subsets for measurement of T cell activation and proliferation were similar to those described above for OT-I CD8^+^ T cells.

### Induction of regulatory T cells

Assessment of intracellular Foxp3 expression was used to identify regulatory T cells. Activated CD4^+^ T cells were prepared as described above. These were stained with antibodies specific to CD4, Vγ2 and Thy1.2 prior to resuspension in 200 μl of Foxp3 Fix/Perm solution (Biolegend) and incubation in the dark for 20 min. Cells were washed once with FACS buffer, followed by washing with Foxp3 Fix/Perm buffer. Supernatant was discarded completely, and cells resuspended in 200 μl Foxp3 Perm buffer (Biolegend). Cells were incubated in the dark for a further 15 min and then centrifuged to remove supernatant. The pellet was resuspended in 20 μl anti-Foxp3-PE conjugate (Biolegend) with incubation in the dark for 30 min. Cells were washed twice with FACS buffer and Foxp3 expression determined by flow cytometric analysis.

### Quantitative polymerase chain reaction

The procedure used follows a similar protocol used previously [24]. L-DC and cDC-like cells were sorted from 28-day co-cultures of Lin^−^ bone marrow over 5G3. RNA was extracted using the RNeasy mini kit (Qiagen, Clifton Hill, Australia) and was converted to cDNA using the RT^2^ First Strand Synthesis Kit (Qiagen). To perform real-time quantitative reverse transcriptase polymerase chain reactions (qRT-PCR), cDNA, RT^2^ SYBR Green Mastermix, and RNAse-free water were added directly to PCR arrays already loaded with primers for 84 genes related to mouse dendritic and antigen presenting cells (PAMM-406A PCR array: SABiosciences, Frederick, MD, USA). The plate was loaded on to a Roche LightCycler 480 (Roche, Castle Hill, Australia). Cycling conditions were: 1 cycle of 10 min at 95 °C, followed by 45 cycles of 15 s at 95 °C, and 1 min at 60 °C. Data analysis was performed using Roche LightCycler 480 software version 1.2.9.11 to calculate the cycle number at which the maximal increase in fluorescence emission occurs in the log-linear phase (threshold cycle C_t_). C_t_ values were calculated for genes of interest (GOI) and housekeeping genes (HKG): ΔC_t_ = C_t_(GOI) – C_t_(HKG). The average ΔC_t_ was taken from duplicate experiments. The fold change difference between two samples was calculated as 2^-ΔCt^ (sample 1)/2^-ΔCt^ (sample 2), giving the relative difference in mRNA quantity between 2 samples for any gene of interest. Technical replicates were averaged for each sample and production of an amplified product validated through gel electrophoresis.

### Measurement of cytokines

The production of IL-2, IL-4, IL-10 and IFN-γ released from activated T cells after incubation with DC subsets for 4 days was measured. A 50 μl aliquot of T cell supernatant was collected and added to the wells of an ELISArray plate containing pre-coated capture antibodies (Qiagen; Victoria, Australia). The plate was incubated on ice for 2 h, followed by washing thrice with 200 μl PBS and centrifugation at 300 g and 4 °C for 5 min. Supernatant was discarded and 100 μl of detection antibody added to each well. The plate was then incubated on ice for a further hour, followed by washing thrice. Supernatant was discarded and 100 μl of avidin*-*horseradish peroxidase (Avidin-HRP) added to each well, followed by incubation on ice for 15 min, and washing thrice. The supernatant was discarded and 100 μl of developing solution added. A_450_ was read using FLUOstar Optima microplate reader (BMG Labtech; Durham, NC, USA)*.* Cytokine production was calculated as A_450_ of sample relative to A_450_ of a standard provided with the kit and expressed as % of maximum.

### Statistical analysis

Statistical analysis involved the pairwise comparison of replicated cultures which were established at the same time, and in some cases assayed at several time points. The statistical procedure used involved a Bonferroni Correction to the significance level of the Student’s *t*-test used to assess significance (*p* ≤ 0.05), reflecting the fact that multiple comparisons were made together. Data are presented as mean ± standard error (SE) for sample size n.

## Abbreviations

APC: antigen presenting cell
cDC: conventional DC
CFSE: carboxyfluorescein diacetate succinimidyl ester
DC: dendritic cell
DCreg: regulatory DC
Lin^−^: lineage-depleted
OVA: ovalbumin
pDC: plasmacytoid DC
PI: propidium iodide
sDMEM: supplemented Dulbecco’s Minimal Esssential Medium
TCR-tg: TCR transgenic
Treg: regulatory T cell

## References

[1] Steinman RM, Banchereau J (2007). Taking dendritic cells into medicine. Nature.

[2] Steinman RM, Hawiger D, Nussenzweig MC (2003). Tolerogenic dendritic cells. Annu Rev Immunol.

[3] Tan JK, O'Neill HC (2005). Maturation requirements for dendritic cells in T cell stimulation leading to tolerance versus immunity. J Leukoc Biol.

[4] Liu J, Cao X (2015). Regulatory dendritic cells in autoimmunity: a comprehensive review. J Autoimmun.

[5] Schmidt SV, Nino-Castro AC, Schultze JL (2012). Regulatory dendritic cells: there is more than just immune activation. Front Immunol.

[6] Morelli AE, Thomson AW (2007). Tolerogenic dendritic cells and the quest for transplant tolerance. Nat Rev Immunol.

[7] Li Q, Guo Z, Xu X, Xia S, Cao X (2008). Pulmonary stromal cells induce the generation of regulatory DC attenuating T-cell-mediated lung inflammation. Eur J Immunol.

[8] Liu Q, Zhang C, Sun A, Zheng Y, Wang L, Cao X (2009). Tumor-educated CD11bhighIalow regulatory dendritic cells suppress T cell response through arginase I. J Immunol.

[9] Tang H, Guo Z, Zhang M, Wang J, Chen G, Cao X (2006). Endothelial stroma programs hematopoietic stem cells to differentiate into regulatory dendritic cells through IL-10. Blood.

[10] Xia S, Guo Z, Xu X, Yi H, Wang Q, Cao X (2008). Hepatic microenvironment programs hematopoietic progenitor differentiation into regulatory dendritic cells, maintaining liver tolerance. Blood.

[11] Zhang M, Tang H, Guo Z, An H, Zhu X, Song W, Guo J, Huang X, Chen T, Wang J (2004). Splenic stroma drives mature dendritic cells to differentiate into regulatory dendritic cells. Nat Immunol.

[12] Svensson M, Kaye PM (2006). Stromal-cell regulation of dendritic-cell differentiation and function. Trends Immunol.

[13] Svensson M, Maroof A, Ato M, Kaye PM (2004). Stromal cells direct local differentiation of regulatory dendritic cells. Immunity.

[14] O'Neill HC, Wilson HL, Quah B, Abbey JL, Despars G, Ni K (2004). Dendritic cell development in long-term spleen stromal cultures. Stem Cells.

[15] O'Neill HC, Griffiths KL, Periasamy P, Hinton RA, Petvises S, Hey YY, Tan JK (2014). Spleen stroma maintains progenitors and supports long-term hematopoiesis. Current Stem Cell Res Ther.

[16] Petvises S, O'Neill HC (2014). Distinct progenitor origin distinguishes a lineage of dendritic-like cells in spleen. Front Immunol.

[17] Tan JK, Ni K, Le F, O'Neill HC (2007). Hematopoiesis of immature myeloid dendritic cells in stroma-dependent spleen long-term cultures occurs independently of NF-KB/RelB function. Exp Hematol.

[18] Tan JK, O’Neill HC (2010). Haematopoietic stem cells in spleen have distinct differentiative potential for antigen presenting cells. J Cell Mol Med.

[19] Wilson HL, Ni K, O’Neill HC (2000). Identification of progenitor cells in long-term spleen stromal cultures that produce immature dendritic cells. Proc Natl Acad Sci U S A.

[20] Despars G, O'Neill HC (2006). Splenic endothelial cell lines support development of dendritic cells from bone marrow. Stem Cells.

[21] Despars G, O'Neill HC (2006). Heterogeneity amongst splenic stromal cell lines which support dendritic cell hematopoiesis. In Vitro Cell Dev Biol Anim.

[22] Periasamy P, Tan JK, Griffiths KL, O'Neill HC (2009). Splenic stromal niches support hematopoiesis of dendritic-like cells from precursors in bone marrow and spleen. Exp Hematol.

[23] Periasamy P, O'Neill HC (2013). Stroma-dependent development of two dendritic-like cell types with distinct antigen presenting capability. Exp Hematol.

[24] Periasamy P, Petvises S, O'Neill HC (2013). Development of two distinct dendritic-like APCs in the context of splenic stroma. Front Immunol.

[25] Petvises S, O'Neill HC (2014). Characterisation of dendritic cells arising from progenitors endogenous to murine spleen. PLoS One.

[26] Hinton RA, O'Neill HC (2012). Technical advance: in vitro production of distinct dendritic-like antigen-presenting cells from self-renewing hematopoietic stem cells. J Leukoc Biol.

[27] Fancke B, Suter M, Hochrein H, O’Keeffe M (2008). M-CSF: a novel plasmacytoid and conventional dendritic cell poietin. Blood.

[28] Shuford WW, Klussman K, Tritchler DD, Loo DT, Chalupny J, Siadak AW, Brown TJ, Emswiler J, Raecho H, Larsen CP (1997). 4-1BB costimulatory signals preferentially induce CD8+ T cell proliferation and lead to the amplification in vivo of cytotoxic T cell responses. J Exp Med.

[29] Matozaki T, Murata Y, Okazawa H, Ohnishi H (2009). Functions and molecular mechanisms of the CD47-SIRPalpha signalling pathway. Trends Cell Biol.

[30] Lund J, Olsen OH, Sorensen ES, Stennicke HR, Petersen HH, Overgaard MT (2013). ADAMDEC1 is a metzincin metalloprotease with dampened proteolytic activity. J Biol Chem.

[31] Sathe P, Shortman K (2008). The steady-state development of splenic dendritic cells. Mucosal Immunol.

[32] Shih VF, Davis-Turak J, Macal M, Huang JQ, Ponomarenko J, Kearns JD, Yu T, Fagerlund R, Asagiri M, Zuniga EI (2012). Control of RelB during dendritic cell activation integrates canonical and noncanonical NF-kappaB pathways. Nat Immunol.

[33] Baranov MV, Ter Beest M, van den Bogaart G. Reaching for far-flung antigen: how solid-core podosomes of dendritic cells transform into protrusive structures. Commun Integr Biol. 2014;7(5) 10.4161/cib.29084 PMC4594491 26843902

[34] Sundararaj S, Zhang J, Krovi SH, Bedel R, Tuttle KD, Veerapen N, Besra GS, Khandokar Y, Praveena T, Le Nours J (2018). Differing roles of CD1d2 and CD1d1 proteins in type I natural killer T cell development and function. Proc Natl Acad Sci U S A.

[35] Seeger P, Musso T, Sozzani S (2015). The TGF-beta superfamily in dendritic cell biology. Cytokine Growth Factor Rev.

[36] Yee NK, Hamerman JA (2013). Beta (2) integrins inhibit TLR responses by regulating NF-kappaB pathway and p38 MAPK activation. Eur J Immunol.

[37] Welner RS, Bararia D, Amabile G, Czibere A, Benoukraf T, Bach C, Wansa KD, Ye M, Zhang H, Iino T (2013). C/EBPalpha is required for development of dendritic cell progenitors. Blood.

[38] Siddiqui S, Schwarz F, Springer S, Khedri Z, Yu H, Deng L, Verhagen A, Naito-Matsui Y, Jiang W, Kim D (2017). Studies on the detection, expression, glycosylation, dimerization, and ligand binding properties of mouse Siglec-E. J Biol Chem.

[39] Corcoran L, Vremec D, Febbraio M, Baldwin T, Handman E (2002). Differential regulation of CD36 expression in antigen-presenting cells: Oct-2 dependence in B lymphocytes but not dendritic cells or macrophages. Int Immunol.

[40] Popa C, van Lieshout AW, Roelofs MF, Geurts-Moespot A, van Riel PL, Calandra T, Sweep FC, Radstake TR (2006). MIF production by dendritic cells is differentially regulated by toll-like receptors and increased during rheumatoid arthritis. Cytokine.

[41] Mackay CR, Sallusto F (2006). A new role for CCR5 in innate immunity--binding to bacterial heat shock protein 70. Eur J Immunol.

[42] Despars G, Ni K, Bouchard A, O'Neill TJ, O'Neill HC (2004). Molecular definition of an in vitro niche for dendritic cell development. Exp Hematol.

[43] Hey YY, O'Neill HC (2016). Antigen presenting properties of a myeloid dendritic-like cell in murine spleen. PLoS One.

[44] Petvises S, Talaulikar D, O'Neill HC (2016). Delineation of a novel dendritic-like subset in human spleen. Cell Mol Immunol.

[45] Hey YY, Tan JK, O'Neill HC (2016). Redefining myeloid cell subsets in murine spleen. Front Immunol.

[46] Papathanasiou P, Petvises S, Hey YY, Perkins AC, O’Neill HC (2017). Impact of the c-MybE308G mutation on mouse myelopoiesis and dendritic cell development. PLoS One.

[47] Auffray C, Fogg DK, Narni-Mancinelli E, Senechal B, Trouillet C, Saederup N, Leemput J, Bigot K, Campisi L, Abitbol M (2009). CX3CR1+ CD115+ CD135+ common macrophage/DC precursors and the role of CX3CR1 in their response to inflammation. J Exp Med.

[48] Fogg DK, Sibon C, Miled C, Jung S, Aucouturier P, Littman DR, Cumano A, Geissmann F (2006). A clonogenic bone marrow progenitor specific for macrophages and dendritic cells. Science.

[49] Liu K, Victora GD, Schwickert TA, Guermonprez P, Meredith MM, Yao K, Chu FF, Randolph GJ, Rudensky AY, Nussenzweig M (2009). In vivo analysis of dendritic cell development and homeostasis. Science.

[50] Onai N, Obata-Onai A, Schmid MA, Ohteki T, Jarrossay D, Manz MG (2007). Identification of clonogenic common Flt3+M-CSFR+ plasmacytoid and conventional dendritic cell progenitors in mouse bone marrow. Nat Immunol.

[51] O’Keeffe M, Fancke B, Hochrein H (2010). The generation of plasmacytoid and conventional dendritic cells with M-CSF. Methods Mol Biol.

